# AI-Based Spatial Burn Assessment with MLLMs: Body Region, 3 × 3 Grid Classification and Burn Instance Counting from Photographs and Semantic Segmentation Masks

**DOI:** 10.3390/biomedicines14091996

**Published:** 2026-09-04

**Authors:** Ibrahim Güler, Armin Kraus, Gerrit Grieb, Henrik Stelling

**Affiliations:** 1 Department of Plastic, Aesthetic and Hand Surgery, Otto-von-Guericke University, 39120 Magdeburg, Germany; armin.kraus@med.ovgu.de; 2 Department of Health Management, Friedrich-Alexander-Universität Erlangen-Nürnberg, 90403 Nuremberg, Germany; henrik.stelling@fau.de; 3 Department of Plastic Surgery and Hand Surgery, Gemeinschaftskrankenhaus Havelhöhe, 14089 Berlin, Germany; gerritgrieb@gmx.de; 4 Department of Plastic Surgery and Hand Surgery, Medical Faculty, Rheinisch-Westfälische Technische Hochschule Aachen, 52074 Aachen, Germany; 5 Practices for Nuclear Medicine, 12157 Berlin, Germany

**Keywords:** burn assessment, spatial localization, image segmentation, clinical photography, multimodal large language model, artificial intelligence, medical imaging, clinical decision support, clinical translation, instance counting

## Abstract

**Background**: Burn photography is a stringent testbed for visual artificial intelligence (AI) in medical imaging, since it permits quantitative and qualitative clinically relevant parameters to be elicited. It is therefore a useful proxy for current vision AI. Reducing a photograph to a semantic segmentation removes color, texture and anatomy while providing pixel-exact boundaries. How spatial assessment responds, and how far instance-level structure can be recovered from a map that does not encode it, is unknown. **Methods**: Three state-of-the-art multimodal large language models (MLLMs) each assessed 153 burn photographs five times under three input conditions: the photograph alone (IMAGE), its segmentation mask alone (MASK), or both combined (BOTH). Four items were elicited per image: burn presence, the burned body region, per-cell classification of a 3 × 3 grid, and the number of separate burns, a surrogate for instance-level recognition. Each model was compared across conditions, paired on the same images. **Results**: Body-region accuracy was 97.1–100.0% under IMAGE, 47.5–54.0% under MASK and 50.2–99.9% under BOTH. Grid-cell accuracy was 77.7–82.3%, 77.9–96.3% and 90.9–98.4%. On the burn count, exact-match was 60.5–66.3%, 61.3–96.6% and 84.7–97.4%, and no model significantly beat the trivial classifier under IMAGE, two of three did under MASK and all three under BOTH. Across 18 paired comparisons of BOTH against a single channel, BOTH was better in 11, worse in three and indistinguishable in four. **Conclusions**: Combining the two inputs improved spatial assessment in most comparisons and degraded it in others, consistently across neither models nor tasks. Additional visual information therefore does not deterministically improve model performance, and disruption remains possible.

## 1. Introduction

A burn is characterized both qualitatively, by features such as its anatomical location and its distribution over the body surface, and quantitatively, by parameters such as the surface area involved (conventionally given as a percentage of total body surface area, TBSA) and the depth reached (termed burn depth or burn degree, graded numerically from first to third or fourth or verbally as superficial, superficial partial thickness, deep partial thickness and full thickness). Which body region is involved bears on the functional and aesthetic outcome to be expected, and thereby on management: burns of the face, hands, feet, genitalia, perineum and major joints raise particular short- and long-term problems, and they also appear among the burn center referral criteria of national and international guidelines. The pattern matters alongside the site and follows from the mechanism, since a single event, whether a burn or a scald, can produce one contiguous area or several separate ones that differ in area and in depth. The presence of multiple separate areas can complicate the estimation of overall burn extent. Both the anatomical location of a burn and whether it is confluent or scattered (multifocal) are therefore integral to its clinical assessment [1,2,3,4,5,6,7,8,9,10,11].

Both quantities are still often obtained in routine practice by long-established rules of thumb, among them the surface-area charts and the rule of nines for TBSA, with depth assigned by visual inspection of the wound. Estimates arrived at in this way vary between individual assessors (inter-rater reliability) and, analogously, between the referring unit and the receiving burn center (inter-center agreement), and since area and depth together inform clinical as well as administrative decisions (transfer to a specialized center versus treatment in-house), that variability carries into management [12,13,14,15,16,17,18].

New technology has usually reached burn care from the specialist end: as dedicated instrumentation in a small number of centers, evaluated first in experimental settings and adopted where infrastructure and trained personnel allowed. General-purpose artificial intelligence (GPAI) has arrived in the opposite order. Building on the large language models (LLMs) that preceded them, contemporary multimodal large language models (MLLMs) take images, video, audio and text as input and return output in those same modalities, require neither local infrastructure nor task-specific training, and are remotely hosted rather than locally deployed, accessed from ordinary consumer devices, from smartphones and tablets to laptops and desktop computers. Whether specialized, scientific and professional applications will ultimately be served by general-purpose systems or by narrow artificial intelligence (NAI) built for a single task is an open question that reaches well beyond medicine. In practice, however, GPAI systems have achieved near-universal distribution through free entry-level tiers and low-cost subscription tiers, and they now constitute a feature of everyday life rather than a prospect, which makes them, in health as in other domains, an object of study in their own right [19,20,21,22,23,24,25].

Most current artificial intelligence (AI) use is accounted for by a small number of proprietary systems: three closed-weight providers together draw the greater part of worldwide consumer and enterprise use of generative artificial intelligence (GenAI) services [26,27]. None of them is released as open-source or open-weight software, and their model weights cannot be deployed locally. What is disclosed about their training data and internal design remains incomplete, and the overall level of disclosure remains low in an ecosystem that drives billions of dollars in investment. Access to sampling parameters such as temperature, top-p, and top-k is generally restricted, in a manner that depends on the model and the interface, with some parameters unavailable or fixed in particular deployment settings. Neither the representation an input receives nor the process by which an answer is arrived at is therefore open to examination. How such a system deals with what it is given is accessible only from the outside, from what it returns when the input is varied, which places the study of these models closer to behavioral than to mechanistic investigation [28,29,30,31,32,33].

**Figure 1:**
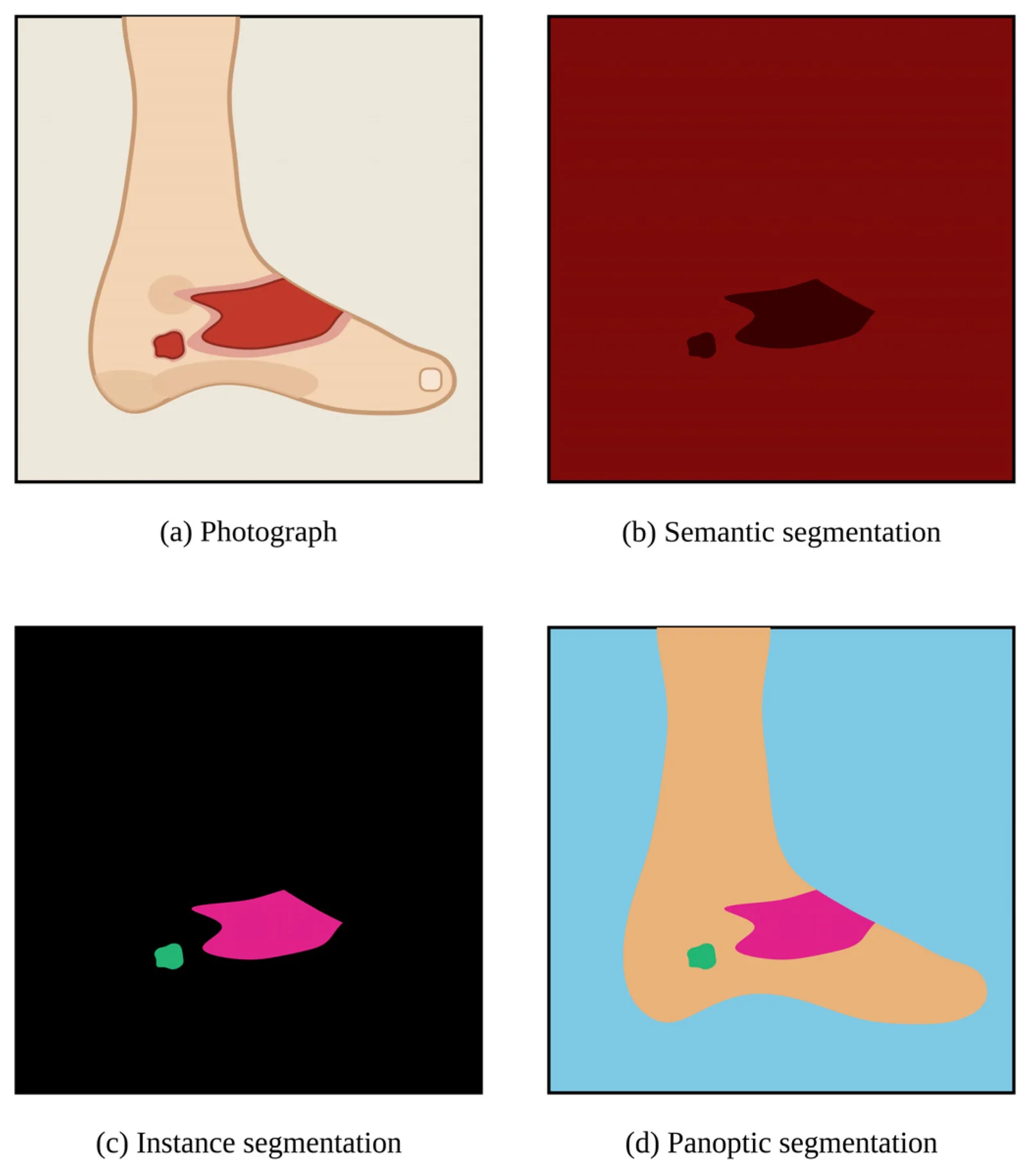
Forms of image segmentation, illustrated on a schematic depiction of a burn injury. (**a**) Photograph, showing two separate burned areas; (**b**) semantic segmentation in the binary form supplied to the models in this study, assigning every pixel to burn or non-burn without separating one burned area from another; (**c**) instance segmentation, separating the two burned areas and leaving all remaining pixels unlabeled; (**d**) panoptic segmentation, which combines the two by assigning every pixel a class label and every burned area an identity of its own. The presentation follows [35,36]. The taxonomy is shown in its general form. The unburned foot therefore appears as a class of its own in (**d**), whereas the binary scheme in (**b**), which is the one used here, distinguishes only burn from non-burn.

Varying the input is one way of approaching that, and the variation can go in either direction, toward more information, toward less, or toward information of a different kind. The clinical photograph is the medium in which burns are already routinely documented, which makes it a practical starting point. Producing a segmentation of such a photograph alters the quality of the information a model receives, and in work on burn photographs using convolutional neural networks (CNNs) segmentation is the established route to the burn area [34]. Unlike image-level classification, which assigns a single label to the whole frame, segmentation labels the individual pixels, and the result is a label map that stands on its own rather than an overlay on the photograph. The form of the labeling determines what that map carries: a semantic segmentation assigns each pixel to a class, in the present setting burn wound or not, irrespective of whether the non-wound pixels show unaffected skin or the wall behind the patient, and without separating one wound from another; an instance segmentation separates individual objects; a panoptic segmentation combines the two (Figure 1) [35,36].

What is lost is everything the labeling does not encode, in a segmentation of a burn wound for example its color, its texture and the surrounding anatomical context, while what remains is the extent of the affected tissue with pixel-exact boundaries. Whether reducing a clinical photograph in this way, or supplementing it with a segmentation mask, whether human-annotated or automatically generated, helps or hinders an artificial intelligence model is not obvious: removing information may discard both task-relevant and task-irrelevant visual cues, while multimodal models do not necessarily ignore irrelevant input [37]. Supplying both at once removes neither, so an input that carries the photograph and its segmentation together might be expected not to perform below either alone. What follows evaluates three state-of-the-art (SOTA) MLLMs on four spatial burn assessment tasks, each item presented as the photograph alone, as its segmentation alone, or as both. The aim is to establish how far the spatial assessment such systems return depends on the form in which the image is supplied, and how far a single answer can be relied on. The study is offered as an approach to these questions rather than a settlement of them.

## 2. Materials and Methods

### 2.1. Study Design

Three SOTA MLLMs were evaluated in a cross-sectional benchmarking design on a retrospectively compiled set of burn photographs and their corresponding semantic segmentation masks, each image presented under three input conditions with five independent inferences per model and condition (multi-run design). Four items were assessed in every run and for every image, with one image constituting one case: whether a burn was visible at all (binary, yes or no), the burned body region, the involvement of each cell of a 3 × 3 grid, and the burn count. The study was designed around the input ablation: each model was compared across the three conditions, paired on the same images. Each model and condition was additionally compared with a trivial classifier, and the models were compared with one another within each condition. Both the Checklist for Artificial Intelligence in Medical Imaging (CLAIM) and the Standards for Reporting Diagnostic Accuracy Studies using Artificial Intelligence (STARD-AI) were considered during preparation. Neither applies in full to a study of this nature and these objects, so no claim of complete adherence is made [38,39].

### 2.2. Dataset

The dataset used here (Extended Burn Images Segmentation; EBIS) has been employed in prior work and is deposited in a public repository [40]. It provides color photographs of burn injuries, acquired under heterogeneous conditions, together with the corresponding semantic segmentation masks, pixel-wise and binary, produced manually with clinical burn expertise. The analysis set comprised 153 photographs and their 153 masks [41,42,43,44,45].

### 2.3. Reference Standards

Burn presence was a binary judgment. Body region was recorded as trunk, head or extremity, being the region in which the burn entirely or predominantly lay. For the grid item, a 3 × 3 grid of nine equal rectangular cells, numbered row-wise from the upper left, was rendered onto each image. Each cell was classified as empty, partial or full. A cell counted as full only if its entire area was burned, as partial if any portion of it was burned, however small, and as empty if none was. The burn count was the number of separate burns, two burns counting as separate whenever unburned tissue lay between them, so that a single injury divided by a strip of intact skin counted as two. The separately counted burns are referred to as burn instances. Reference values were established by two physicians in a consensus procedure following the instruction given to the models, with the photograph and the segmentation mask considered together.

### 2.4. Models and Inference Protocol

Three SOTA MLLMs were evaluated, the designation denoting here the most recent general-purpose flagship model each provider had released to its consumer web interface at the time of data collection, one from each of the three closed-weight providers that together account for the greater part of worldwide consumer and enterprise use of generative AI [26,27]. Selection was by provider and release status and did not draw on benchmark results or leaderboard positions. The models were Gemini 3.1 Pro (Google DeepMind, London, UK, and Mountain View, CA, USA), GPT-5.6 Sol (OpenAI, San Francisco, CA, USA) and Claude Fable 5 (Anthropic, San Francisco, CA, USA). All three were accessed through their first-party web interfaces in July 2026 under default settings, without use of an application programming interface (API) and without configuration or customization of any kind, that is without fine-tuning and without system prompting or priming with in-context examples. These interfaces expose no temperature or related sampling controls.

Under IMAGE the photograph was the single visual input. Under MASK it was the corresponding semantic segmentation mask, a two-tone image in which one tone marks the burned and the other the unburned pixels (schematically illustrated in Figure 1b). Under BOTH the photograph and its corresponding mask formed the two panels of a single composite image, in a fixed arrangement and format across all 153 cases. Composing them into one image holds the number of visual inputs at one across all three conditions. The grid was rendered onto each panel.

Every image was queried five times in each condition, each run an independent request in a new context, yielding 6885 requests and 82,620 item responses in total (153 images × 3 conditions × 3 models × 5 runs × 12 items). The prompt was identical throughout, except for the description of the supplied image as IMAGE, MASK or BOTH.

### 2.5. Outcome Measures

Burn detection is reported descriptively as the detection rate, the reference being positive for all 153 images. For body region the outcomes were accuracy and the distribution of predicted classes. The answers given under BOTH were additionally compared with those given under each single channel. Because both series draw on the same three classes, the observed agreement is set against the agreement expected if the two were independent, given how often each class was answered in each. This is agreement between answers, not accuracy.

For the grid the spatial question is whether a cell contains burned tissue, so the primary analysis is the binary collapse into empty versus burned cells, with partial and full counted together as burned. Outcomes were accuracy, sensitivity, specificity and the grid-cell Jaccard index, with the intersection over union computed over grid cells rather than pixels: cells called burned by both the model and the reference, divided by cells called burned by either, per image and then averaged. Unlike accuracy it disregards cells that both call empty. Accuracy by grid position is reported descriptively. The secondary ternary analysis reports the distribution of predicted classes together with per-class recall (sensitivity) and precision (positive predictive value), macro-averaged F1 and balanced accuracy, computed at the cell level over all five runs. The full class holds 18 of the 1377 reference cells, so the two class-averaged measures weight it equally with two classes that are far larger, and they are read alongside the per-class values rather than on their own. For the burn count the outcomes were the mean absolute error (MAE), that is the per-image absolute error averaged over images and runs, and the exact-match rate.

Each model and condition was also compared with a trivial classifier returning the most frequent reference class for each item: extremity for body region, burned for every grid cell, one burn for the burn count.

Inter-run reliability (IRR), also termed inter-run reproducibility, is the agreement among the five repeated queries rather than with the reference, and was quantified by Fleiss’ κ, unweighted for body region and for the binary grid collapse at the cell level and quadratically weighted on the ternary grid scale, by the intraclass correlation coefficient ICC(A,1) for the burn count, and by the percentage of items answered identically in all five runs. For burn detection κ is undefined, one answer category being used almost exclusively, and the mean pairwise agreement among the five runs is given instead [46,47,48,49].

Metrics computed within each run were averaged over the five runs. Fleiss’ κ, mean pairwise agreement, ICC(A,1) and the percentage answered identically in all five runs are by definition quantities across runs. For the binary hypothesis tests the runs were reduced to one answer per item, the modal answer for categorical items with ties resolved in favor of the earliest run and the median for the burn count, giving majority-answer accuracy and median-answer exact match. Every test therefore operates on one value per image: the reduced answer for body region and for the exact match of the burn count, the run-averaged per-image proportion of correctly classified cells for the grid, and the run-averaged per-image absolute error where the burn count is analyzed as a continuous outcome. Since every image contributes the same 45 cell judgments, the grid quantity entering the tests is numerically equal to the cell-level accuracy reported for that task. There the aggregation fixes the unit of analysis rather than the value of the estimate. Both the run-averaged and the reduced value are reported for body region and for the burn count. Ties can arise only for body region, the only item with more than two categories and one answer per run. The median of five counts cannot tie, and the grid is not reduced to a modal answer at all. The tie rule was examined by resolving ties by the latest instead of the earliest run and by the resolutions least and most favorable to the model.

### 2.6. Statistical Analysis

Confidence intervals (CIs) are percentile intervals from a case-level non-parametric bootstrap with 2000 resamples of the 153 images at a fixed seed. Resampling at the image level preserves the dependence among the five runs and among the nine cells of one image. Grid descriptives are reported at the cell level, except the grid-cell Jaccard index, which is image-level by construction, and every grid test uses the per-image proportion of correctly classified cells.

Pairwise comparisons were performed only where the omnibus test was significant. The comparisons with the trivial classifier are planned single comparisons and have no omnibus. For binary outcomes the omnibus was Cochran’s Q and the pairwise test the exact McNemar test. For per-image continuous outcomes, the proportion of correctly classified cells and the per-image absolute error of the burn count, they were the Friedman and Wilcoxon signed-rank tests. The Benjamini–Hochberg false discovery rate procedure was applied within each family, a family being one task on one axis, and q is reported as the decision value, with *p* given for the omnibus tests [50]. Effect sizes are in original units and matched to the test, the risk difference in percentage points (pp) for McNemar and the median per-image paired difference for Wilcoxon, each with a bootstrap interval, and the number of images differing in each direction is reported alongside. The significance level was two-sided α = 0.05. All analyses were performed in Python 3.12 (Python Software Foundation, Wilmington, DE, USA) with standard statistical and data visualization libraries.

## 3. Results

### 3.1. Reference Standard Distributions and Burn Detection

All 82,620 item responses entered the analysis. None was missing, invalid or excluded. The reference body region was extremity in 79 of 153 images (51.6%), trunk in 53 (34.6%) and head in 21 (13.7%). Of the 1377 grid cells (153 images × 9 cells), 420 were empty (30.5%), 939 partial (68.2%) and 18 full (1.3%), so 957 cells (69.5%) were burned in the binary collapse. The share burned varied by position, from 49.7% in the upper right cell to 98.7% in the center cell, with all four corner cells below 60%. The reference burn count had a median of 1 (interquartile range 1 to 2) and a range of 1 to 11, with 98 of 153 images (64.1%) containing exactly one burn. The corresponding trivial classifiers therefore attain 51.6% on body region, 69.5% of cells on the grid task and 64.1% on the burn count.

A burn was present in all 153 images, so burn detection is degenerate and no discrimination can be measured. Of 6885 detection responses, two (0.03%) returned “no”, both under IMAGE and both from Gemini 3.1 Pro, giving a detection rate of 99.7% for that model and condition and 100.0% for every other combination. Mean pairwise agreement across the five runs was 99.48% and 100.00% correspondingly. No response declared a burn visible while returning zero separate burns.

### 3.2. Body Region

**Figure 2:**
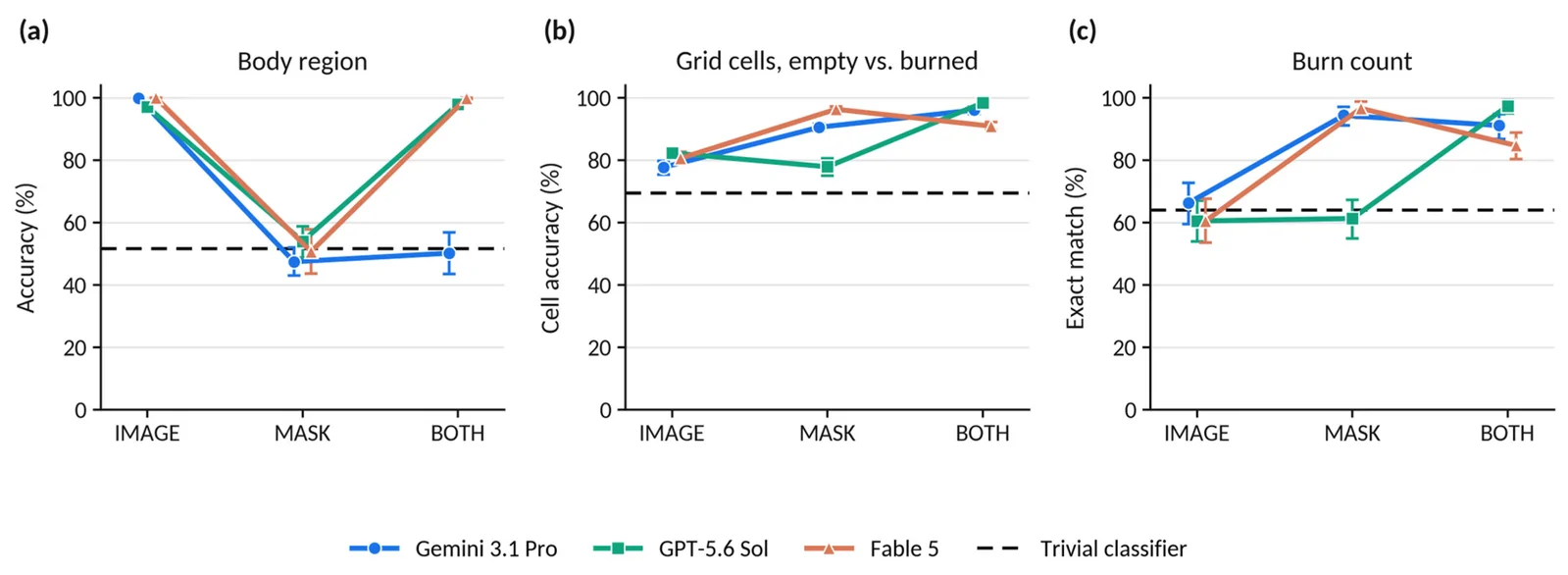
Performance across the three input conditions for each model and task. (**a**) Body-region accuracy; (**b**) accuracy of the 3 × 3 grid classification in the binary collapse, empty versus burned cells, at the cell level; (**c**) exact-match rate for the burn count. Values are computed within each of the five runs and then averaged; error bars are 95% case-level bootstrap confidence intervals. The three input conditions were applied to the same 153 images, and the connecting lines mark this pairing. The dashed line is the trivial classifier that returns the most frequent reference class on every item, at 51.6% in (**a**), 69.5% of cells in (**b**) and 64.1% in (**c**).

Under IMAGE all three models were at or near ceiling, with run-averaged accuracies of 99.9% (95% CI 99.6 to 100.0) for Gemini 3.1 Pro, 97.1% (95.4 to 98.6) for GPT-5.6 Sol and 100.0% (100.0 to 100.0) for Fable 5 (Table 1; Figure 2a). Each exceeded the trivial classifier by 47.7 to 48.4 pp on the majority answer (all q < 0.001; Table 2).

Under MASK the three models fell to 47.5% (43.0 to 52.0), 54.0% (48.8 to 58.8) and 50.7% (43.7 to 57.8). On the majority answer none was distinguishable from the trivial classifier, the differences being +1.3 pp for Gemini 3.1 Pro (q = 1.000), +9.8 pp for GPT-5.6 Sol (q = 0.115) and −2.6 pp for Fable 5 (q = 0.986). Fable 5 never returned the head class under MASK, assigning all 765 responses to trunk (570) or extremity (195).

Under BOTH the models separated. GPT-5.6 Sol and Fable 5 returned to 97.9% (96.2 to 99.2) and 99.9% (99.6 to 100.0), neither differing from its own IMAGE value on the majority answer (+0.0 pp, q = 1.000 for both; Table 3, Panel A). Gemini 3.1 Pro reached 50.2% (43.5 to 56.9), a decrease of 47.7 pp relative to IMAGE on the majority answer (0 of 153 images better, 73 worse; q < 0.001), and its majority answer was correct for 52.3% of images, +0.7 pp relative to the trivial classifier (q = 1.000).

**Figure 3:**
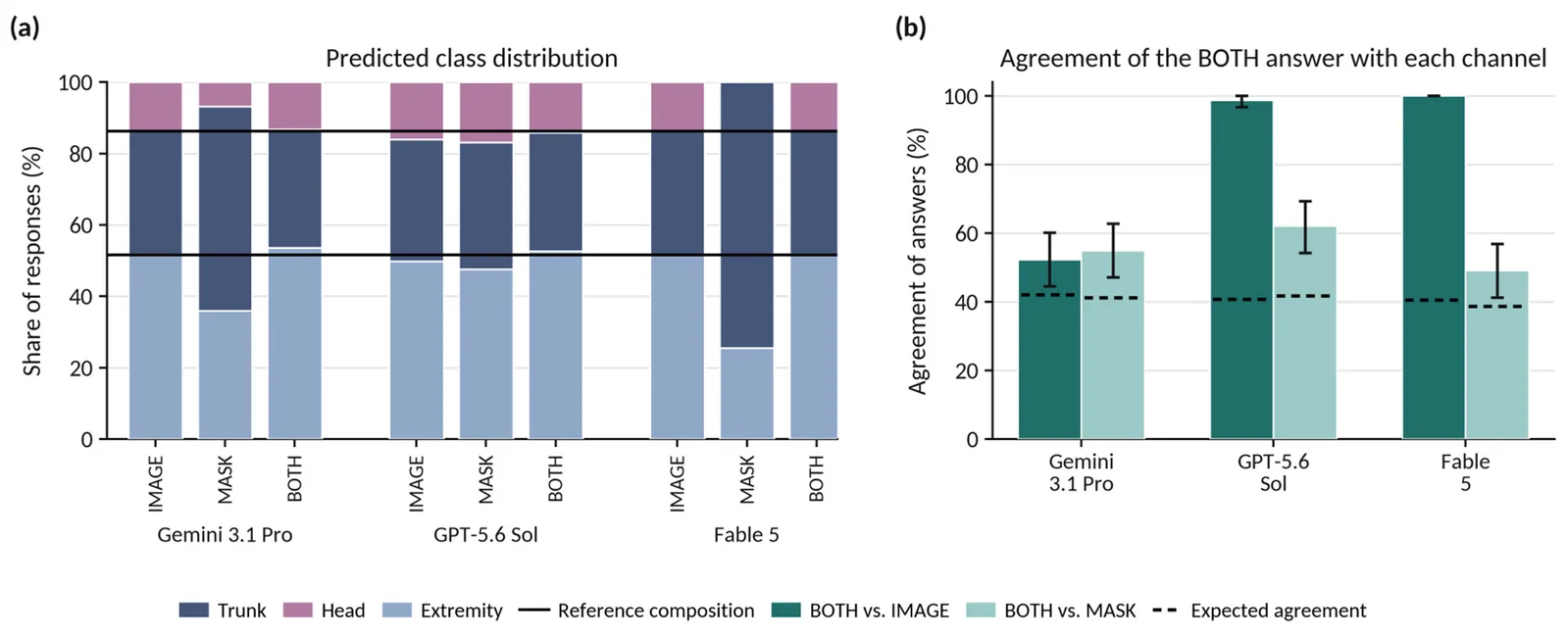
Body-region classification under the three input conditions. (**a**) Distribution of predicted classes as a share of the 765 responses given by each model in each condition. The two solid horizontal lines mark the cumulative composition of the reference standard, at 51.6% extremity and a further 34.6% trunk. (**b**) Agreement of the answer given under BOTH with the answer given under each single input channel, after reducing the five runs of each condition to the modal answer per image. The dashed lines mark the agreement expected if the two sets of answers were independent with the observed marginal class frequencies. Error bars are 95% case-level bootstrap confidence intervals. Agreement of the BOTH answer with the IMAGE answer approaches the accuracy attained under BOTH for models whose IMAGE answers were almost always correct.

The predicted class distribution of Gemini 3.1 Pro under BOTH remained close to the reference composition, with 254 trunk, 101 head and 410 extremity responses against reference frequencies of 265, 105 and 395 (Figure 3a), while per-class sensitivity was 43.4% for trunk, 25.7% for head and 61.3% for extremity. Its answers under BOTH agreed with its own answers under MASK for 54.9% of images (47.1 to 62.7), against an agreement of 41.2% expected under independence, and with its answers under IMAGE for 52.3% (44.4 to 60.1) against 42.0% expected (Figure 3b). On 39 of 153 images (25.5%) the BOTH answer matched neither channel. The corresponding agreement with the MASK answers was 62.1% (54.2 to 69.3) for GPT-5.6 Sol and 49.0% (41.2 to 56.9) for Fable 5, and with the IMAGE answers 98.7% and 100.0%. The latter two values are arithmetically close to the accuracy of those models under BOTH, since their IMAGE answers were almost always correct. Modal ties occurred in 34 of the 1377 image-level reductions of the body-region answer (153 images × 3 models × 3 conditions; 2.5%), all under MASK, 16 for Gemini 3.1 Pro and 18 for GPT-5.6 Sol. Resolving them by the latest instead of the earliest run left every significance decision on this item unchanged, as did the resolution least favorable to the models. Under the resolution most favorable to them one decision changed, GPT-5.6 Sol under MASK against the trivial classifier, at q = 0.115 as reported and q = 0.009 at that extreme.

### 3.3. Grid Cells

Run-averaged cell accuracy in the binary collapse was 77.7% (75.4 to 79.8) for Gemini 3.1 Pro, 82.3% (80.5 to 84.2) for GPT-5.6 Sol and 80.6% (78.7 to 82.6) for Fable 5 under IMAGE; 90.5% (89.4 to 91.6), 77.9% (75.0 to 80.6) and 96.3% (95.4 to 97.2) under MASK; and 96.1% (94.8 to 97.2), 98.4% (97.8 to 98.9) and 90.9% (89.5 to 92.3) under BOTH (Table 1; Figure 2b). All nine model-by-condition comparisons against the trivial classifier were significant, with median per-image differences of +0.0 to +33.3 pp (all q < 0.001). The grid-cell Jaccard index, which disregards cells that both the model and the reference call empty, was 69.7% to 74.9% under IMAGE, 73.8% to 94.2% under MASK and 86.0% to 97.6% under BOTH. Under IMAGE it exceeded the trivial classifier, which attains 69.5% on both metrics because it calls every cell burned, by only 0.2 to 5.4 pp, whereas cell accuracy exceeded the same comparator by 8.2 to 12.8 pp, the difference following from how the two metrics are constructed.

On the per-image proportion of correctly classified cells, the omnibus test was significant for every model (all *p* < 0.001) and the direction of the pairwise comparisons differed between models (Table 3, Panel B). For Gemini 3.1 Pro all three comparisons were positive: MASK exceeded IMAGE by a median paired difference of +11.1 pp (+8.9 to +13.3), BOTH exceeded IMAGE by +17.8 pp (+15.6 to +17.8) and BOTH exceeded MASK by +6.7 pp (+4.4 to +6.7), all q < 0.001. For GPT-5.6 Sol, MASK was below IMAGE by −2.2 pp (−6.7 to +0.0; 59 images better, 80 worse, 14 unchanged; q = 0.010), while BOTH exceeded IMAGE by +15.6 pp and MASK by +17.8 pp (both q < 0.001). For Fable 5, MASK exceeded IMAGE by +15.6 pp and BOTH exceeded IMAGE by +8.9 pp (both q < 0.001), but BOTH was below MASK by −4.4 pp (−4.4 to −2.2; 13 images better, 100 worse, 40 unchanged; q < 0.001).

**Figure 4:**
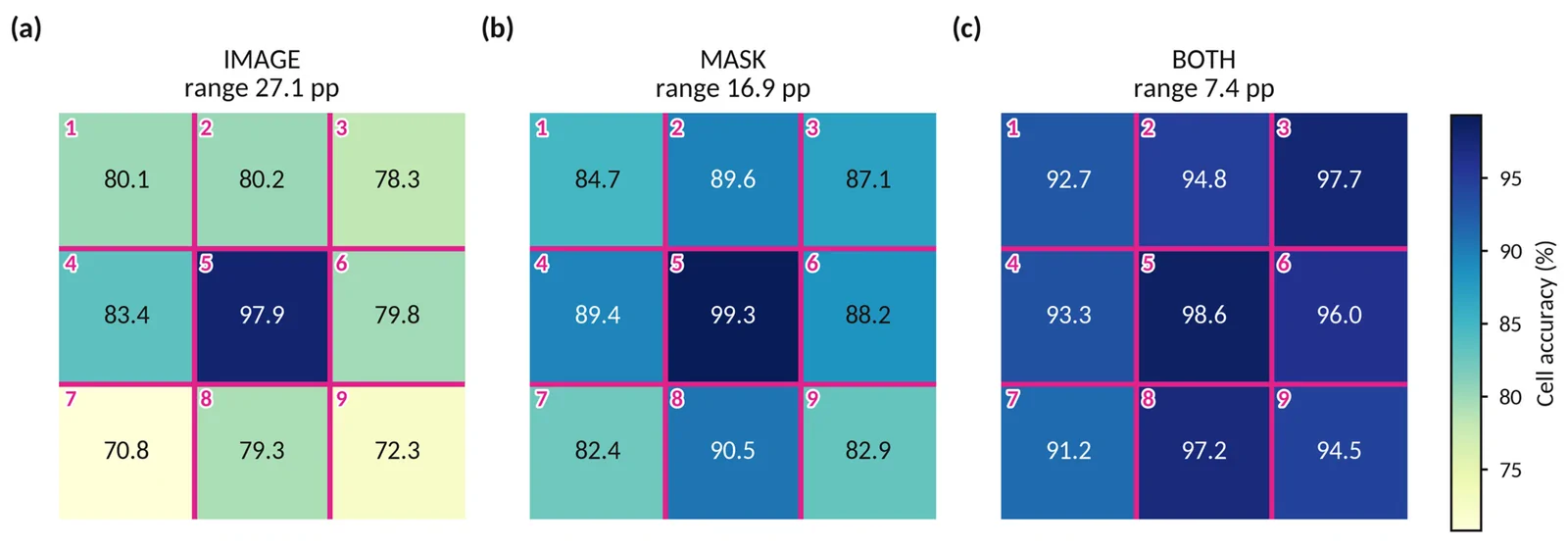
Accuracy of the 3 × 3 grid classification by position in the grid, in the binary collapse of empty versus burned cells, pooled over the three models. (**a**) IMAGE; (**b**) MASK; (**c**) BOTH. Each square carries the cell number in magenta, numbered row-wise from the upper left as in the stimulus, and at its center the run-averaged percentage of correctly classified instances of that grid position across the 153 images and five runs. The nine positions are shown in their spatial arrangement within the image frame. The range printed above each panel is the difference between the highest and the lowest of the nine positions. pp, percentage points.

Accuracy varied with position in the grid, and the variation narrowed across the three conditions (Figure 4). Pooled over the three models, the center cell was classified correctly in 97.9% of instances under IMAGE, 99.3% under MASK and 98.6% under BOTH, while the weakest position in every condition, the lower left cell, reached 70.8%, 82.4% and 91.2%. The range across the nine positions was 27.1 pp under IMAGE, 16.9 pp under MASK and 7.4 pp under BOTH.

The composition of the errors also changed. Of 6885 cell responses per model and condition, the ratio of false positives to false negatives under IMAGE was 0.70, 0.56 and 0.75 for Gemini 3.1 Pro, GPT-5.6 Sol and Fable 5, and under MASK 1.87, 1.01 and 0.12. Under BOTH the false positives fell to 13, 23 and 42 while the false negatives stood at 257, 88 and 585, for ratios of 0.05, 0.26 and 0.07.

**Figure 5:**
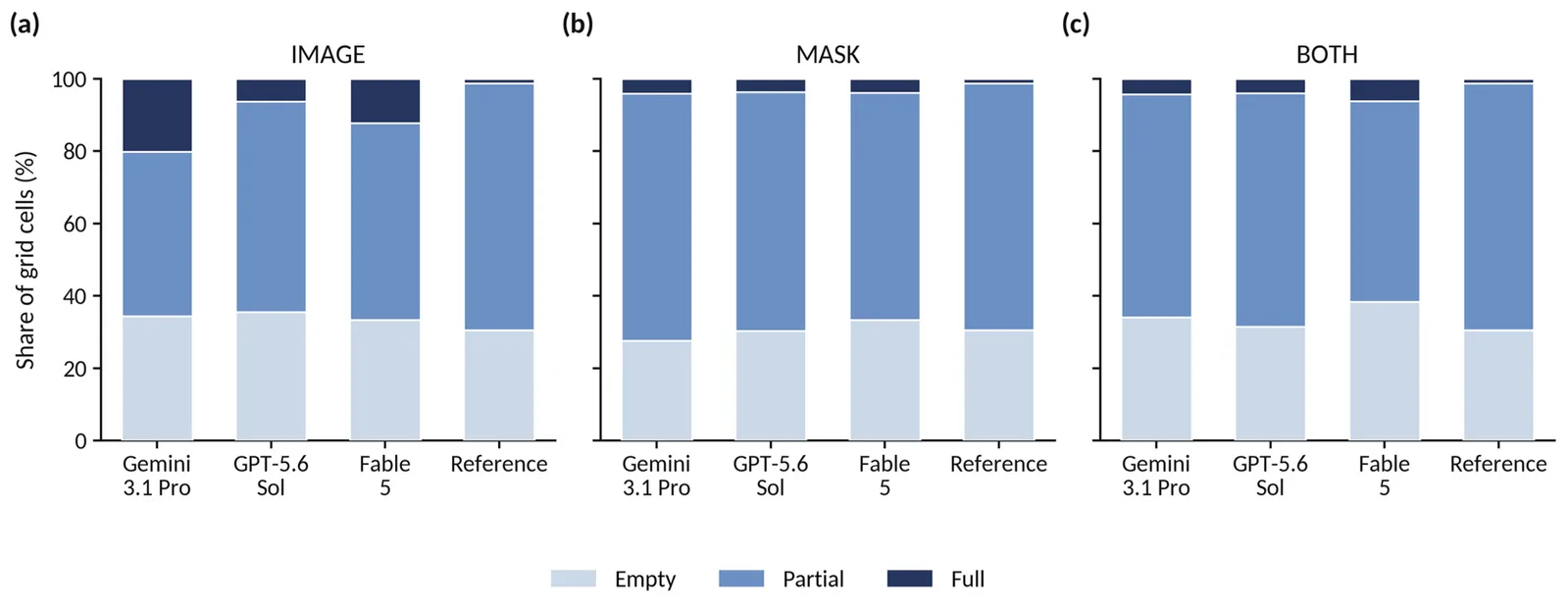
Distribution of predicted classes on the ternary grid scale, as a share of the 6885 cell responses given by each model in each condition, against the distribution of the 1377 reference cells. (**a**) IMAGE; (**b**) MASK; (**c**) BOTH. Full denotes a cell burned in its entirety. The reference bar is identical in all three panels and is repeated for comparison.

In the secondary ternary analysis the full class, which accounts for 1.3% of reference cells, was assigned to 20.1% of cells by Gemini 3.1 Pro, 6.3% by GPT-5.6 Sol and 12.2% by Fable 5 under IMAGE, a factor of up to 15.4 (Figure 5). Under MASK the corresponding shares were 4.1%, 3.7% and 3.9%, and under BOTH 4.3%, 4.0% and 6.1%. The shares assigned to the empty class ranged from 27.6% to 38.4% across all nine combinations, against a reference share of 30.5%. Under IMAGE they were 34.4%, 35.6% and 33.3%, so both outer classes exceeded their reference shares in that condition. On the same scale, per-class recall was 70.0% to 79.3% for empty, 54.2% to 75.4% for partial and 58.9% to 70.0% for full under IMAGE; 63.5% to 98.7%, 79.5% to 91.4% and 53.3% to 100.0% under MASK; and 98.0% to 99.4%, 80.3% to 94.2% and 87.8% to 100.0% under BOTH. Precision, in the same class order, was 62.0% to 68.0%, 81.3% to 88.5% and 4.5% to 12.2% under IMAGE; 63.8% to 90.2%, 82.2% to 99.4% and 18.9% to 33.3% under MASK; and 77.9% to 95.9%, 98.7% to 99.8% and 18.7% to 32.5% under BOTH. Macro-averaged F1 was 46.4% to 58.3%, 57.5% to 79.8% and 68.7% to 81.1%, and balanced accuracy 64.7% to 71.2%, 65.4% to 96.7% and 88.7% to 97.7%.

### 3.4. Burn Instance Count

Under IMAGE the median-answer exact-match rate was 68.0% for Gemini 3.1 Pro, 61.4% for GPT-5.6 Sol and 61.4% for Fable 5, against 64.1% for the trivial classifier that always answers one burn. None of the three differed from it, the risk differences being +3.9 pp (q = 0.231), −2.6 pp (q = 0.541) and −2.6 pp (q = 0.597), with the point estimate below the trivial classifier for two of the three (Table 2). Run-averaged MAE was 0.677 burns (0.505 to 0.871), 0.833 (0.651 to 1.030) and 0.699 (0.531 to 0.889), against 0.771 for the trivial classifier (Table 1; Figure 2c), and the models under-counted more often than they over-counted, in 28.2%, 25.1% and 27.2% of responses against 5.5%, 14.4% and 12.3%.

Under MASK the models divided. Gemini 3.1 Pro and Fable 5 reached median-answer exact-match rates of 95.4% and 97.4%, exceeding the trivial classifier by 31.4 pp and 33.3 pp (both q < 0.001), with MAE falling to 0.067 (0.031 to 0.108) and 0.034 (0.012 to 0.064). GPT-5.6 Sol reached 56.2%, a difference of −7.8 pp from the trivial classifier (q = 0.121), with an MAE of 0.875 burns (0.667 to 1.112), and did not differ from its own IMAGE performance (−5.2 pp, q = 0.340; Table 3, Panel C).

Under BOTH all three exceeded the trivial classifier, at median-answer exact-match rates of 94.1%, 98.7% and 86.9% and risk differences of +30.1, +34.6 and +22.9 pp (all q < 0.001). Relative to their own single-channel conditions, GPT-5.6 Sol improved under BOTH over both IMAGE (+37.3 pp) and MASK (+42.5 pp, both q < 0.001), Gemini 3.1 Pro did not differ from MASK (−1.3 pp, q = 0.625), and Fable 5 fell below MASK by 10.5 pp (2 images better, 18 worse; q < 0.001). A supporting analysis of the per-image absolute error reaches the same conclusion as the exact-match result in eight of the nine comparisons and diverges in one: Gemini 3.1 Pro showed a larger absolute error under BOTH than under MASK on 20 images against four in the opposite direction (q = 0.008), at a median paired difference of 0.00 burns, where the exact-match outcome showed no difference.

### 3.5. Inter-Run Reliability

**Figure 6:**
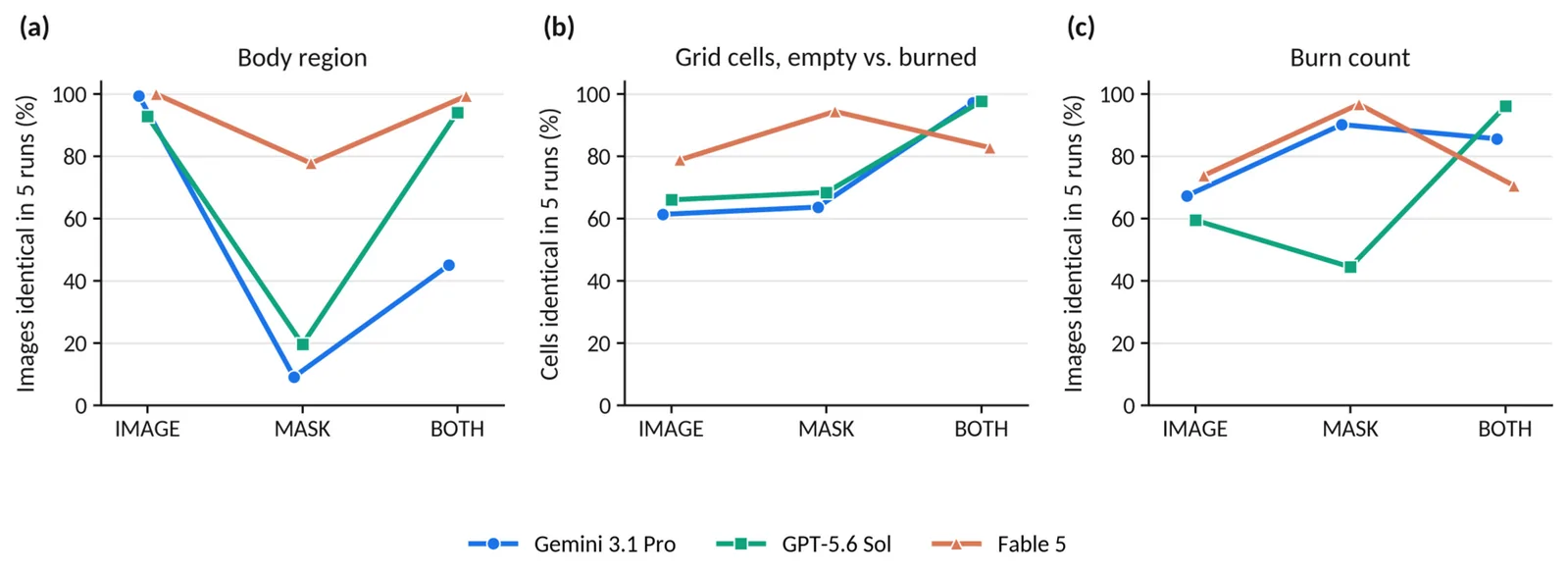
Inter-run reliability across the five repeated queries, expressed as the percentage of items for which all five runs returned the same answer. (**a**) Body region, per image; (**b**) grid classification in the binary collapse, per cell; (**c**) burn count, per image. The quantities plotted describe agreement among the five runs of one model on one item and do not describe agreement with the reference standard; they are reported as point estimates in Table 4. The three input conditions were applied to the same 153 images, and the connecting lines mark this pairing.

IRR across the five repeated queries varied with the input condition more than with the task (Table 4; Figure 6). On body region, the proportion of images answered identically in all five runs was 99.3%, 92.8% and 100.0% under IMAGE for Gemini 3.1 Pro, GPT-5.6 Sol and Fable 5, with Fleiss’ κ of 1.00 (0.99 to 1.00), 0.93 (0.89 to 0.97) and 1.00 (1.00 to 1.00). Under MASK the same proportions were 9.2%, 19.6% and 77.8%, with κ of 0.07 (0.03 to 0.11), 0.28 (0.22 to 0.34) and 0.67 (0.56 to 0.76). Under BOTH they were 45.1%, 94.1% and 99.3%, with κ of 0.62 (0.57 to 0.68), 0.96 (0.93 to 0.98) and 1.00 (0.99 to 1.00). For Gemini 3.1 Pro, BOTH fell between the two single channels rather than tracking either, at 45.1% against 99.3% under IMAGE and 9.2% under MASK. For the other two models BOTH returned to the level of their IMAGE values.

IRR and correctness did not coincide. Fable 5 under MASK returned the same body region in all five runs for 77.8% of images at a Fleiss’ κ of 0.67, while its majority answer differed from the trivial classifier by −2.6 pp and was not distinguishable from it (q = 0.986).

In one of the five runs of Gemini 3.1 Pro under MASK the model returned trunk for all 153 images and classified all 1377 grid cells as burned. That run is constant on body region and on the binary collapse of the grid, and contributes no discrimination on either.

On the grid task at the cell level, the proportion of cells answered identically in all five runs was 61.4%, 66.0% and 78.9% under IMAGE, 63.7%, 68.4% and 94.4% under MASK and 97.2%, 97.7% and 82.8% under BOTH, with binary Fleiss’ κ from 0.58 to 0.97. Quadratically weighted Fleiss’ κ on the ternary scale ranged from 0.59 to 0.98 across the nine combinations. For the burn count, ICC(A,1) ranged from 0.47 (0.21 to 0.67) for Gemini 3.1 Pro under IMAGE to 1.00 (0.99 to 1.00) for Fable 5 under MASK, and the proportion of images answered identically in all five runs from 44.4% for GPT-5.6 Sol under MASK to 96.7% for Fable 5 under MASK.

### 3.6. Overall Pattern of the Input Ablation and Comparison Between Models

Across the three tasks the ablation yielded 18 paired comparisons of BOTH against a single input channel, three models by three tasks by two channels. BOTH was significantly better in 11 of these, significantly worse in three and not distinguishable in four, and the three deteriorations occurred in two of the three models (Table 3). The omnibus test was significant in all nine model-by-task cells of this axis, so no pairwise comparison was withheld.

In the comparison between models, each difference being the later minus the earlier model in the order Gemini 3.1 Pro, GPT-5.6 Sol and Fable 5, the three were not distinguishable in three of the nine condition-by-task combinations tested, the omnibus test being non-significant for body region under IMAGE (*p* = 0.368) and under MASK (*p* = 0.054) and for the burn count under IMAGE (*p* = 0.072). No pairwise comparisons were performed in those cells. Where the models did differ, the largest differences were under BOTH on body region, where GPT-5.6 Sol and Fable 5 exceeded Gemini 3.1 Pro by 47.1 pp and 47.7 pp (both q < 0.001) while not differing from each other (+0.7 pp, q = 1.000). On the grid task all three pairwise comparisons were significant under MASK and under BOTH, with median paired differences from −8.9 to +17.8 pp. Under IMAGE two of three reached significance, at +2.2 pp. On the burn count under MASK, GPT-5.6 Sol was 39.2 pp below Gemini 3.1 Pro and 41.2 pp below Fable 5 (both q < 0.001), which did not differ from each other (+2.0 pp, q = 0.453). Under BOTH the three differed pairwise by −11.8 to +4.6 pp.

## 4. Discussion

Three SOTA MLLMs were evaluated on four spatial burn assessment tasks under a three-way input ablation. Alongside the photograph, the second visual channel was the binary semantic segmentation mask of that same photograph, separating burned from unburned pixels. What can be derived from such a mask is fixed by what it encodes. Which grid cells contain burned tissue, and how many separate burns are present, are properties of the geometry it represents, whereas the body region is not, because a map that distinguishes only burn from non-burn holds no anatomy. Since the combined condition contains everything either single channel contains, no model should on that reading perform worse under it than under either alone. The expectation treats an added input as neutral at worst and leaves aside the possibility that a second panel competes for attention. Two of the three models violated it, on different tasks, while the third met it throughout. Where the mask was suited to the question the gains were usually large, though one model did not benefit from it without the photograph alongside. Where it could not carry the answer, adding it to the photograph left two models unaffected and reduced the third to trivial-classifier level, with the photograph still in the input. In these data, whether an additional visual channel helps, does nothing or degrades performance thus depended on the model rather than on the information supplied.

### 4.1. Segmentation as a Reduction of the Photograph

A segmentation replaces the photograph rather than annotating it. Color, texture and anatomical context are gone, and what remains is the extent of the affected tissue with pixel-exact boundaries. What is traded is one kind of information for another rather than more information for less, and whether the trade pays depends on the kind the question needs.

Setting aside the degenerate detection item, two of the remaining three items ask only about geometry, and for those the mask carries what the question needs. Supplying the mask raised run-averaged cell accuracy under BOTH by 10 to 18 pp and the run-averaged exact-match rate on the burn count by 24 to 37 pp, in every model. This is the straightforward case, with the added channel paying off wherever it accompanied the photograph.

Assigning burns to a 3 × 3 grid is included here as a probe of spatial grounding rather than as a clinical one. It asks whether the model can relate burned tissue to a visible reference frame, which is a precondition for any spatial statement it might make about a photograph. Read that way, the distribution of errors over the nine positions is informative. The center cell was classified correctly in 97.9% to 99.3% of cases in every condition, whereas the weakest position ranged from 70.8% to 91.2%, and across IMAGE, MASK and BOTH the spread across positions contracted from 27.1 to 16.9 to 7.4 pp. It is thus mainly the periphery that the mask channel repairs. A center cell that is burned in 98.7% of reference images can be answered correctly without inspecting the image, simply by always calling it burned. The corners cannot, and it is there that grounding in the image is actually tested. Because the cell boundaries were drawn onto the image, the positional deficit cannot be attributed to the model placing them incorrectly, which leaves a perceptual account of the errors at the edge of the frame. Deficits in basic visual and spatial competence, of which the present pattern is an instance, are widely reported for multimodal models on tasks that people solve without difficulty [31,32].

Under BOTH almost every remaining error was a burned cell called empty, at false-positive to false-negative ratios of 0.05 to 0.26, where under IMAGE they had been 0.56 to 0.75 and under MASK as high as 1.87. The one-sidedness is a property of the input condition rather than a stable trait of any model: wherever the photograph was present all three models erred in the same direction, whereas with the mask alone they did not. Independently of accuracy, a systematic error in either direction carries consequences of its own, under-calling toward under-diagnosis and over-calling toward the reverse, with the corresponding effects on triage and treatment. Those effects lie outside what this study examines. The present data show that the direction of the error moved with the input supplied.

The ternary analysis shows the same shift on a different axis. The full class holds 1.3% of reference cells and was assigned to as many as 20.1% of cells under IMAGE, dropping to at most 6.1% once the mask was available. Inflation of the rarest category occurred in every model and condition, and it took the form of over-assignment: precision on that class was 4.5% to 33.3% across the nine combinations, against a per-class recall of 53.3% to 100.0%. Since a cell counts as full only when no unburned tissue remains in it, the inflation is a perceptual result and not a matter of where a threshold was placed, and it is largest where only the photograph was available: the mask marks the boundary in two tones, whereas on a photograph the same judgment rests on a gradual transition in color and texture. Where that transition is the only cue, the models left the middle category more often, and in both directions rather than in one. This pattern contrasts with the central-tendency compression reported for MLLMs on ordinal clinical scales, where extreme ratings are systematically shifted toward intermediate categories [51].

### 4.2. Modality Interference Between Two Visual Channels, or an Effect of the Composite Frame

The body-region item is the one task in the set that a binary mask cannot answer, and it therefore does double duty. Under MASK it is a control: the three models fell to 47.5–54.0%, none distinguishable from a trivial classifier that always answers extremity. That is the design working, and it argues at the same time against an alternative reading of the mask channel, namely that the outline of the segmented area might itself betray the body part.

The control condition shows how a model behaves when the channel it is given does not carry the information the question requires. In the combined condition that same channel is added alongside one that does. GPT-5.6 Sol and Fable 5 answered as they had from the photograph alone, their accuracy on the majority answer unchanged. In Gemini 3.1 Pro the majority answer was correct for 52.3% of images, 0.7 pp from the trivial classifier, and the photograph was unchanged and still present.

The obvious reading, that Gemini 3.1 Pro simply answered from the mask instead, does not survive the data. Its BOTH answers agreed with its own MASK answers for 54.9% of images, above the 41.2% expected under independence. On a quarter of the images the BOTH answer matched neither channel: the model followed neither. Nor was the ability abolished, with per-class sensitivities of 43.4% for trunk, 25.7% for head and 61.3% for extremity. In aggregate the answers still looked plausible, at 254 trunk, 101 head and 410 extremity responses against reference frequencies of 265, 105 and 395, but a distribution that close is what drawing at random from the same class proportions would produce as well, and per image the answers were correct for 52.3% against the 42.0% such drawing would deliver. What survived was the naming of classes in roughly the right numbers. What degraded was their assignment to cases, and only the second is of any use at the bedside.

The inability of an MLLM to disregard a channel that is irrelevant to the question has been described as modality interference, for example between text and image, with irrelevant text degrading image tasks and the reverse [37]. The constellation here is image against image, two panels of one visual input, one of them informative for the question asked and one not. Where the construction of such a model is disclosed, a visual encoder is coupled to an LLM through a projection layer. When that encoder is a vision transformer (ViT), it divides the image into patches, embeds each of them with its position, and relates every patch to every other by self-attention across the whole frame. The systems evaluated here are closed, so it is not known whether a composite frame is separated into its two panels before encoding or carried through as a single image, nor whether models of the same general construction handle it alike. That the same three models differ so sharply is consistent with the handling of a composite frame differing between systems, without establishing that it does. On this evidence the failure can be attributed to the individual system but not to the model class, and a single system passing the same test would not settle the question for the class either [37,52].

The mechanism cannot be identified here, since that would require interpretability work on systems whose weights, training data and internal design are not disclosed, and the wording matters accordingly. To a human observer the combined condition is the same photograph with a mask beside it. To the model it is a different input, and in how many respects it differs cannot be determined from the outside. On the aforementioned behavioral reading [30], adding the mask demonstrably disrupted an unrelated judgment in one of three models, but the spatial content of the mask cannot be identified as the cause. Both channels were supplied in a single composite frame, so what these data establish is susceptibility to that presentation, with interference between the two channels a candidate explanation rather than a demonstrated one (Section 4.5).

### 4.3. Division of Labor Between Model Components

Beyond what these tasks report about the models evaluated, the setting raises a design question that is not specific to burns. Automated burn assessment has commonly been built from task-trained networks, in some cases more than one per system [53,54,55]. General-purpose models have also been applied directly to burn photographs without an explicit segmentation step [56,57]. The conditions used here represent a different experimental scenario. A general-purpose model was given a delineation it did not produce, and one that was not model output at all but the annotation supplied with the dataset. The mask conditions therefore describe a favorable case rather than a workflow, since no segmenter would deliver exactly that. Even at that ceiling one model could not use the delineation on its own, which is the first thing such an arrangement would have to survive.

In a hypothetical but conceivable scenario, a photograph of a burn arrives from a general practitioner, an emergency department or a wound care nurse with a general question attached, whether the injury is severe and whether it needs referral. Such a question could be routed internally rather than answered in one pass: one component reading the appearance of the wound for burn depth, another producing a segmentation from the same photograph so that extent and boundaries can be derived from it, for instance as a contribution to TBSA, and the separate paths meeting again for a joint assessment. The two could draw on different machinery, such as a transformer-based image encoder for one and CNNs, long established for segmentation, for the other. Conceptually this follows the idea of a mixture-of-experts (MoE) or mixture-of-modality-experts (MoME) design [58]. What the input is turned into along the way would then be part of the system rather than something the user supplies. Such an arrangement presupposes a step before any of this, in which a system establishes what it has been given and what would be worth deriving from it, whether a segmentation would help at all and, if so, of what kind, before an integrated assessment is formed. The conditions used here were not designed to test or report that step, since both channels arrived together and were assembled externally, which is not how an input would arise in use. They do show that a delineation can be available and still contribute nothing to the question asked.

### 4.4. Stability and Correctness as Separate Properties

Across the models and conditions examined here, agreement between the five runs of one model, reported here as IRR, varied widely. That variation is worth a separate look, because accuracy is measured against a reference standard and in use there is none, so whether a given answer can be relied on has to be settled without one. Repeating the same query is one of the few checks available in that situation, and it reports something accuracy does not [59].

Both failure modes matter, and they matter differently. Given the same case, the same input and the same question, a system that keeps changing its answer is difficult to use whatever its average accuracy over a series of cases, since in real-world use the model is asked once, and that one answer is what a decision rests on. Repeating the query is a departure from that, and it helps only up to a point: a system that always returns the same answer is easier to work with, but the answer it repeats may just as well be wrong. The second is the harder to notice, since repetition reads to a user as confirmation and offers a confidence the system has not earned. Stability, of which IRR is one measure, and correctness are therefore separate properties, and neither substitutes for the other. In these data one model answered a question consistently while performing at trivial-classifier level. What repetition cannot do is indicate which of several stable answers is right.

### 4.5. Limitations

The first limitation follows from a design decision. Presenting two inputs, in this case two images, separately and then in combination requires a form for the combination that adds as little of its own as possible. Two separate uploads, or supplying the second image in a following turn, would each have introduced asymmetries, so BOTH was created by merging IMAGE and MASK horizontally into one frame with no further modification. The process neither added nor removed anything: BOTH is IMAGE placed next to MASK at their original resolution and without recompression, with no border, background or label added and no element that either condition did not already contain. What happens to that frame afterwards is not under the same control, since a visual encoder may impose a fixed budget in pixels, patches or vision tokens, in which case the composite would be scaled to it as a whole. Such a reduction would be expected to affect the fine-grained spatial items first, whereas both of those improved under BOTH relative to the photograph alone in every model and it was the coarse anatomical judgment that failed, in one model only. How the frame is taken up cannot be established from the outside with closed systems (Section 4.2).

The degradation observed under BOTH could therefore arise late, in how the two sources are weighted against one another once both have been read, or early, in how the composite is taken to begin with, if it is resolved into two images at all. These data cannot distinguish the two, and no control condition with an uninformative second panel, such as the photograph presented twice or a blank mask, was collected. Either way the observation matters for the arrangement sketched in Section 4.3, since a routing component directing a photograph to specialized vision modules and recombining what they return presupposes that the recombination is itself unproblematic.

The burn count is a surrogate for instance-level recognition, in this setting the identification of individual burns. The term burn instance is used throughout as a label for the separately counted areas as defined in Section 2.3, and not as a claim that the models placed individual burns in correspondence with the reference. The count shows that a model arrived at the right number of separate areas, not that it identified the same ones, and the right number can follow from a combination of errors, one burn overlooked while another is split in two. Testing that directly would require the models to produce a segmentation of their own rather than a number: semantic, assigning every pixel a class, instance, assigning every burned area an identity, or panoptic, doing both (Figure 1). Ideally such output would be returned as an image or failing that as a textual assignment of every pixel to a class and, where applicable, to an instance. Neither appears practical at present in a form that could be standardized across cases, across repeated runs and across models from different vendors.

Burn detection is degenerate, since the reference is positive for every image and no negative cases exist. Specificity cannot be computed, so the reported rate can at most be read as a sensitivity, and it cannot separate a model that recognizes a burn from one that answers affirmatively whatever it is shown. Under MASK an affirmative answer may in addition rest on the format itself: segmentation masks of burns are available on the internet and may have entered training corpora, and they share characteristics that are recognizable as such, being two-valued, hard-edged and textureless. A model may identify the format and answer that a burn is present without extracting anything from the particular image before it. Beyond the format, contamination of the training data cannot be excluded for the dataset as a whole, neither with the photographs of the individual cases nor with the segmentation masks, both of which are available in a public repository [40].

Reference-standard uncertainty is a structural rather than incidental feature of medical AI evaluation, since clinical categories are often imperfectly defined and expert readers disagree [60]. Here it takes a specific form. The reference standard was established by two physicians reading jointly and simultaneously, so no independent rating series exists and neither inter-rater nor intra-rater reliability could be quantified. The reference standard is therefore reported without an estimate of its own precision, which matters here because visual assessment of burns is known to vary between assessors [12,13,14,15], and every accuracy figure in this study carries that unquantified component. Blinded independent double reading and a blinded re-reading after an interval would be reasonable approaches to this.

Some features of the study design constrain the analysis further. The nine cells of one image are not independent observations, since they share the acquisition conditions and the model’s reading of that image. Grid descriptives are therefore reported at the cell level while all grid tests use the per-image proportion of correctly classified cells, so that each image contributes a single observation. The 3 × 3 grid is itself a compromise: a coarser 2 × 2 or a finer 5 × 5 division would have shifted what the task demands, and no sample size calculation was performed for either the number of images or the number of cells. The analysis set was not sized against a target precision or a prespecified effect, so the confidence intervals reported here describe the precision obtained rather than a precision planned for. Because all four items were elicited simultaneously, coupling between the answers cannot be excluded. This was accepted deliberately: consecutive prompts, splitting the items across separate queries, or chain-of-thought (CoT) approaches would each have reduced comparability further, between runs of the same model as well as between models. A single query, one message and no follow-up, appeared to be the best available compromise for standardization.

The evaluation uses one photograph per case, without clinical context such as age, mechanism or time since injury, and without the multiple views or examination available at the bedside. The images were acquired under heterogeneous illumination, at unrecorded and presumably varying times since injury, and with different cameras and camera-to-subject distances, none of which could be controlled or analyzed as a covariate, so every estimate reported here is pooled over that heterogeneity rather than conditioned on it. Skin type is likewise neither documented nor analyzed, although both the appearance of a burn and the contrast between burned and unburned tissue depend on it, which bears in particular on the judgments made from the photograph alone. Confidence estimates were elicited neither from the models nor from the raters establishing the reference standard, so the certainty attached to an answer was not assessed alongside the stability of that answer across runs (Section 4.4). Where such estimates have been collected in a diagnostic imaging setting, discrepancies between a model’s stated confidence and the correctness of its answer have been described [61].

Although both were considered during the design of the study, neither CLAIM nor STARD-AI applies in full. Several item groups of these reporting guidelines have no counterpart in a study of this kind, among them participant recruitment and setting, a clinical reference standard, a participant flow diagram, indeterminate results, and a defined intended use [38,39]. Finally, three models at one point in time are a narrow sample of a fast-moving field. Each was queried through its consumer web interface, chosen deliberately because that is how these systems are used by the great majority of their users, which means that sampling behavior is set by the vendor and the served version may change without notice [62,63].

## 5. Conclusions

The MLLMs evaluated here are closed systems, and what they do with an input can be established only from the outside, by changing the input and observing what comes back. The same photograph and the same questions were put with a segmentation mask alongside it, in place of it, or not at all, and what came back did not follow from the information the input carried. In three of 18 paired comparisons the combined input was worse than a single channel, and the same addition of the mask that raised one model to near-perfect counting reduced another, on an unrelated judgment, to trivial-classifier level with the photograph still in front of it. Whether an added channel is taken up without cost to an unrelated judgment was, here, a property of the system rather than of the task, and no single accuracy figure reports it.

That matters most for a technology that reached clinical users from the consumer end rather than the specialist one, where safety depends on whoever asks the model. Safety presupposes understanding, and that means knowing how a model treats content that is relevant or irrelevant to the question asked, and on what basis it prioritizes between them. What was quantified here is behavior and not mechanism, and both will need to be worked out and understood.

## Figures and Tables

**Table 1:** Task performance under the three input conditions.

Model	Cond.	Body Region		Grid Cells, Empty vs. Burned				Burn Count		
		Acc., %	Maj., %	Acc., %	Sens., %	Spec., %	Jacc., %	MAE	Exact, %	Med., %
Gemini 3.1 Pro	IMAGE	99.9 (99.6–100.0)	100.0	77.7 (75.4–79.8)	81.1	70.0	69.7	0.677 (0.505–0.871)	66.3 (59.5–72.7)	68.0
Gemini 3.1 Pro	MASK	47.5 (43.0–52.0)	52.9	90.5 (89.4–91.6)	95.3	79.8	88.5	0.067 (0.031–0.108)	94.4 (91.1–97.1)	95.4
Gemini 3.1 Pro	BOTH	50.2 (43.5–56.9)	52.3	96.1 (94.8–97.2)	94.6	99.4	93.4	0.101 (0.058–0.149)	91.1 (86.8–94.8)	94.1
GPT-5.6 Sol	IMAGE	97.1 (95.4–98.6)	99.3	82.3 (80.5–84.2)	83.6	79.3	74.9	0.833 (0.651–1.030)	60.5 (54.0–67.1)	61.4
GPT-5.6 Sol	MASK	54.0 (48.8–58.8)	61.4	77.9 (75.0–80.6)	84.2	63.5	73.8	0.875 (0.667–1.112)	61.3 (54.9–67.3)	56.2
GPT-5.6 Sol	BOTH	97.9 (96.2–99.2)	99.3	98.4 (97.8–98.9)	98.2	98.9	97.6	0.027 (0.009–0.052)	97.4 (95.0–99.2)	98.7
Fable 5	IMAGE	100.0 (100.0–100.0)	100.0	80.6 (78.7–82.6)	84.0	72.9	73.2	0.699 (0.531–0.889)	60.5 (53.6–67.6)	61.4
Fable 5	MASK	50.7 (43.7–57.8)	49.0	96.3 (95.4–97.2)	95.3	98.7	94.2	0.034 (0.012–0.064)	96.6 (93.6–98.8)	97.4
Fable 5	BOTH	99.9 (99.6–100.0)	100.0	90.9 (89.5–92.3)	87.8	98.0	86.0	0.231 (0.156–0.318)	84.7 (80.4–88.9)	86.9
Trivial classifier	—	51.6	51.6	69.5	100.0	0.0	69.5	0.771	64.1	64.1

Acc. = accuracy; Maj. = majority answer; Sens. = sensitivity; Spec. = specificity; MAE = mean absolute error, in burn instances; Med. = median answer. *n* = 153 images per model and condition, comprising 1377 grid cells. Intervals in parentheses are 95% CIs. Majority-answer and median-answer values are computed across the 5 runs; all other values are run-averaged. Jacc., % = grid-cell Jaccard index, computed per image and averaged over images and runs; it disregards cells that both call empty. Trivial classifier = always extremity, always burned, always one instance.

**Table 2:** Comparison with the trivial classifier for body region and the burn count.

Model	Cond.	Body Region			Burn Count		
		*n* +/−	Risk Difference, pp	q	*n* +/−	Risk Difference, pp	q
Gemini 3.1 Pro	IMAGE	74/0	+48.4 (+40.5 to +56.2)	<0.001	10/4	+3.9 (−0.7 to +9.2)	0.231
GPT-5.6 Sol	IMAGE	73/0	+47.7 (+39.9 to +55.6)	<0.001	7/11	−2.6 (−7.8 to +2.6)	0.541
Fable 5	IMAGE	74/0	+48.4 (+40.5 to +56.2)	<0.001	14/18	−2.6 (−9.8 to +5.2)	0.597
Gemini 3.1 Pro	MASK	48/46	+1.3 (−11.1 to +13.1)	1.000	48/0	+31.4 (+24.2 to +39.2)	<0.001
GPT-5.6 Sol	MASK	39/24	+9.8 (−0.7 to +19.0)	0.115	14/26	−7.8 (−15.0 to +0.0)	0.121
Fable 5	MASK	49/53	−2.6 (−15.0 to +10.5)	0.986	53/2	+33.3 (+25.5 to +41.2)	<0.001
Gemini 3.1 Pro	BOTH	30/29	+0.7 (−9.2 to +10.5)	1.000	48/2	+30.1 (+22.9 to +37.9)	<0.001
GPT-5.6 Sol	BOTH	73/0	+47.7 (+39.9 to +55.6)	<0.001	53/0	+34.6 (+27.5 to +42.5)	<0.001
Fable 5	BOTH	74/0	+48.4 (+40.5 to +56.2)	<0.001	36/1	+22.9 (+16.3 to +30.1)	<0.001

*n* +/− = images correct for the model but not for the trivial classifier, and the reverse; pp = percentage points. Exact McNemar test on the reduced single answer; a positive risk difference favors the model. Benjamini–Hochberg correction within each task, 9 comparisons per family.

**Table 3:** Input-ablation comparisons within each model.

Panel A. Body region; majority answer			
Model	MASK vs. IMAGE	BOTH vs. IMAGE	BOTH vs. MASK
Gemini 3.1 Pro	−47.1 (−54.9 to −39.2), 0/72, <0.001	−47.7 (−55.6 to −39.9), 0/73, <0.001	−0.7 (−10.5 to +9.2), 30/31, 1.000
GPT-5.6 Sol	−37.9 (−45.8 to −30.7), 0/58, <0.001	+0.0 (−2.0 to +2.0), 1/1, 1.000	+37.9 (+30.7 to +45.8), 58/0, <0.001
Fable 5	−51.0 (−58.8 to −43.1), 0/78, <0.001	+0.0 (+0.0 to +0.0), 0/0, 1.000	+51.0 (+43.1 to +58.8), 78/0, <0.001
**Panel B. Grid cells; per-image proportion of correct cells**			
**Model**	**MASK vs. IMAGE**	**BOTH vs. IMAGE**	**BOTH vs. MASK**
Gemini 3.1 Pro	+11.1 (+8.9 to +13.3), 123/20/10, <0.001	+17.8 (+15.6 to +17.8), 136/7/10, <0.001	+6.7 (+4.4 to +6.7), 111/13/29, <0.001
GPT-5.6 Sol	−2.2 (−6.7 to +0.0), 59/80/14, 0.010	+15.6 (+13.3 to +17.8), 135/2/16, <0.001	+17.8 (+13.3 to +22.2), 117/3/33, <0.001
Fable 5	+15.6 (+11.1 to +20.0), 130/7/16, <0.001	+8.9 (+4.4 to +11.1), 111/24/18, <0.001	−4.4 (−4.4 to −2.2), 13/100/40, <0.001
**Panel C. Burn count; exact match of the median answer**			
**Model**	**MASK vs. IMAGE**	**BOTH vs. IMAGE**	**BOTH vs. MASK**
Gemini 3.1 Pro	+27.5 (+20.9 to +34.6), 42/0, <0.001	+26.1 (+19.0 to +33.3), 42/2, <0.001	−1.3 (−3.9 to +1.3), 1/3, 0.625
GPT-5.6 Sol	−5.2 (−13.7 to +3.3), 19/27, 0.340	+37.3 (+29.4 to +44.4), 57/0, <0.001	+42.5 (+34.6 to +50.3), 65/0, <0.001
Fable 5	+35.9 (+27.5 to +43.8), 57/2, <0.001	+25.5 (+18.3 to +32.7), 40/1, <0.001	−10.5 (−16.3 to −5.2), 2/18, <0.001

Each cell gives the effect size (95% CI), the number of images better/worse (Panel B: better/worse/tied), and q. Each comparison is the first condition minus the second. Effect sizes are risk differences in pp (Panels A and C, exact McNemar test) or median per-image paired differences in pp (Panel B, Wilcoxon signed-rank test). Benjamini–Hochberg correction within each panel. Omnibus tests preceded all comparisons, each df = 2 and all *p* < 0.001: Cochran’s Q = 102.08, 114.03, 156.00 (Panel A) and 74.84, 89.74, 80.03 (Panel C); Friedman χ^2^ = 189.19, 169.33, 167.36 (Panel B), in table order. Per-image cell accuracy takes values in multiples of 1/45, so a median difference of 0.0 pp can accompany a significant test; the image counts show how many changed.

**Table 4:** Inter-run reliability (IRR) across the five repeated queries.

Model	Cond.	Body Region		Grid Cells		Burn Count	
		Fleiss’ κ	Ident., %	Fleiss’ κ	Ident., %	ICC(A,1)	Ident., %
Gemini 3.1 Pro	IMAGE	1.00 (0.99–1.00)	99.3	0.58 (0.53–0.62)	61.4	0.47 (0.21–0.67)	67.3
Gemini 3.1 Pro	MASK	0.07 (0.03–0.11)	9.2	0.62 (0.60–0.64)	63.7	0.98 (0.97–0.99)	90.2
Gemini 3.1 Pro	BOTH	0.62 (0.57–0.68)	45.1	0.97 (0.97–0.98)	97.2	0.98 (0.95–0.99)	85.6
GPT-5.6 Sol	IMAGE	0.93 (0.89–0.97)	92.8	0.64 (0.60–0.67)	66.0	0.64 (0.33–0.81)	59.5
GPT-5.6 Sol	MASK	0.28 (0.22–0.34)	19.6	0.68 (0.64–0.72)	68.4	0.56 (0.39–0.66)	44.4
GPT-5.6 Sol	BOTH	0.96 (0.93–0.98)	94.1	0.97 (0.96–0.98)	97.7	0.99 (0.99–1.00)	96.1
Fable 5	IMAGE	1.00 (1.00–1.00)	100.0	0.77 (0.74–0.79)	78.9	0.92 (0.81–0.95)	73.9
Fable 5	MASK	0.67 (0.56–0.76)	77.8	0.93 (0.91–0.94)	94.4	1.00 (0.99–1.00)	96.7
Fable 5	BOTH	1.00 (0.99–1.00)	99.3	0.83 (0.80–0.85)	82.8	0.87 (0.71–0.93)	70.6

Ident. = items answered identically in all 5 runs, over images for body region and burn count and over cells for the grid task; ICC = intraclass correlation coefficient. All values describe agreement among the 5 runs of one model, not agreement with the reference. Fleiss’ κ is unweighted on the 3 nominal body-region classes and on the binary grid collapse. Both grid columns refer to that binary collapse. ICC(A,1) is a two-way random-effects absolute-agreement coefficient for single measurements.

## Data Availability

The dataset used in this study is publicly accessible. Repository details are given in [40].

## Abbreviations

The following abbreviations are used in this manuscript:

AI	artificial intelligence
API	application programming interface
CI	confidence interval
CLAIM	Checklist for Artificial Intelligence in Medical Imaging
CNN	convolutional neural network
CoT	chain-of-thought
GenAI	generative artificial intelligence
GPAI	general-purpose artificial intelligence
ICC	intraclass correlation coefficient
IRR	inter-run reliability (inter-run reproducibility)
LLM	large language model
MAE	mean absolute error
MLLM	multimodal large language model
MoE	mixture-of-experts
MoME	mixture-of-modality-experts
NAI	narrow artificial intelligence
pp	percentage points
SOTA	state-of-the-art
STARD-AI	Standards for Reporting Diagnostic Accuracy Studies using Artificial Intelligence
TBSA	total body surface area
ViT	vision transformer
